# Efficacy of Switching Therapy From Alglucosidase Alfa to Avalglucosidase Alfa on Respiratory Function in Participants With Late‐Onset Pompe Disease: A Post Hoc Analysis From the COMET Trial

**DOI:** 10.1002/jmd2.70033

**Published:** 2025-08-12

**Authors:** Priya S. Kishnani, Matthias Boentert, Stephan Wenninger, Kenneth I. Berger, Jérôme Msihid, Lasair O'Callaghan, Rachida Essadi‐Addou, Victor Gallego, Neeraj Singh Rawat, Olivier Huynh‐Ba, Jordi Diaz‐Manera

**Affiliations:** ^1^ Division of Medical Genetics, Department of Pediatrics Duke University Medical Center Durham North Carolina USA; ^2^ Department of Neurology Institute of Translational Neurology, Münster University Hospital Münster Germany; ^3^ LMU Clinic, Department of Neurology Friedrich‐Baur‐Institute, University of Munich Munich Germany; ^4^ Sanofi Cambridge Massachusetts USA; ^5^ Sanofi Gentilly France; ^6^ Sanofi Madrid Spain; ^7^ Sanofi Hyderabad India; ^8^ John Walton Muscular Dystrophy Research Centre Newcastle University Centre for Life Newcastle upon Tyne UK; ^9^ Neuromuscular Diseases Unit, Neurology Department Hospital de la Santa Creu i Sant Pau Barcelona Spain; ^10^ Centro de Investigación Biomédica en Red en Enfermedades Raras (CIBERER) Madrid Spain

**Keywords:** alglucosidase alfa, avalglucosidase alfa, forced vital capacity, Pompe disease, respiratory outcomes

## Abstract

Pompe disease (PD) is a rare, autosomal recessive neuromuscular disorder caused by a deficiency in acid α‐glucosidase. In the Phase 3 COMET trial (NCT02782741), respiratory function was examined in participants with late‐onset PD randomized to receive enzyme replacement therapy with alglucosidase alfa (ALG; standard of care) or avalglucosidase alfa (AVA; intervention) in the primary analysis period (PAP). The open‐label extended treatment period (ETP) provided long‐term data on AVA treatment. This post hoc analysis evaluated respiratory outcomes in participants who received ALG in the PAP (Weeks 0–49) and switched to AVA in the ETP (Weeks 49–145). Participants were categorized according to improvement in upright forced vital capacity percent‐predicted (FVC) at Week 49. Forty‐four participants were included, of whom 20 (45.5%) had ΔFVC > 0% predicted at Week 49. Among ΔFVC > 0% predicted participants, FVC improved during the PAP (estimated slope [SE]: 4.0 (1.1) %/year, *p* < 0.01), and was maintained following switch to AVA in the ETP (0.1 (0.8) %/year, *p* = 0.86). For participants with ΔFVC ≤ 0% predicted during the PAP (*n* = 24; estimated slope [SE]: −3.4 (1.0) %/year, *p* < 0.01), FVC stabilized following switch to AVA (0.3 (0.7) %/year, *p* = 0.70). Slopes in measures of respiratory airflow and inspiratory/expiratory muscle strength were consistent with these findings. Similar results were observed using ΔFVC ≥ 3% predicted as a measure of clinically meaningful change. This analysis demonstrates clinically meaningful maintenance in measures of lung volume (FVC) and airflow (forced expiratory volume in 1 s) after switching therapy from ALG to AVA that persists for ≥ 2 years and is independent of prior outcomes with ALG.

**Summary**

- This research report describes the long‐term clinical benefits on respiratory function of switching from alglucosidase alfa to avalglucosidase alfa in adults with late‐onset Pompe disease.

## 1 Introduction

Pompe disease (PD) is a rare, autosomal recessive neuromuscular disorder caused by a deficiency in acid α‐glucosidase (GAA) [1, 2, 3], an enzyme that degrades lysosomal glycogen [4]. This deficiency leads to the progressive accumulation of glycogen in various tissues, causing cellular dysfunction, muscular damage, and functional disabilities [1, 2]. Respiratory muscle dysfunction and failure are commonly observed in patients with PD [5].

Enzyme replacement therapy (ERT) with alglucosidase alfa (ALG) has been the standard of care in PD since 2006 [1, 6], and has improved outcomes by reducing lysosomal accumulation of glycogen and slowing disease progression [1]. However, the disease can eventually progress in people with PD after several years of treatment with ALG [1, 7], and several studies have demonstrated variability in individual responses to ERT [7, 8]. Multiple factors may impact responses to ERT, including age and extent of disease progression when starting therapy [4, 9, 10]. While treatment guidelines serve as a resource, the heterogenous nature of PD means it is important to individualize care [7]. Thus, there remains an unmet need in the management of PD [1]. Due to the progressive nature of PD and the linkage between decline in lung function to development of respiratory failure, respiratory function stability can be considered a positive outcome [5].

Avalglucosidase alfa (AVA) is an ERT specifically designed for enhanced targeting of the mannose‐6‐phosphate (M6P) receptor‐mediated uptake, the essential pathway for lysosomal cellular uptake and trafficking [6]. Increased bis‐M6P residues results in increased delivery of the drug to the muscle cells and improved glycogen clearance compared with ALG [1, 6, 11].

In the Phase 3 COMET trial (NCT02782741) [12], respiratory function was examined in participants with late‐onset PD (LOPD) who were randomized to receive either ALG or AVA in the primary analysis period (PAP). Treatment with ALG resulted in a least‐squares (LS) mean (standard error [SE]) improvement in upright forced vital capacity percent‐predicted (FVC) of 0.5% (0.9) at Week 49 [6]. In the open‐label extended treatment period (ETP), LS mean change (SE) in FVC from study baseline was 1.2% (1.3) for the patients switching from ALG to AVA [13].

Here, we report analyses that capitalized on the availability of comprehensive respiratory function data pre‐ and post‐switch from ALG to AVA in COMET. Specifically, the objective of this analysis was to examine respiratory function outcomes in patients who switched from ALG during the PAP to AVA during the ETP, categorized by lung function outcomes while receiving ALG.

## 2 Methods

### 2.1 Study Design and Participants

The study design and eligibility criteria for COMET have been published [6, 12]. Briefly, participants were randomized to receive either ALG or AVA in the PAP, and those that entered the open‐label ETP were all treated with AVA. The present post hoc analysis assessed participants who switched therapy from ALG in the PAP (up to Week 49) to AVA in the ETP (Weeks 49–145); Week 145 of the ETP was considered as the last visit for sample size consideration. The cut‐off date for data used in this analysis was 31 May 2023, corresponding to the study completion.

### 2.2 Outcomes Assessed

Respiratory outcomes assessed in this post hoc analysis included: respiratory function measured by FVC in the upright position, forced expiratory volume in 1 s (FEV1), maximal inspiratory pressure (MIP), and maximal expiratory pressure (MEP). All data for the respiratory outcomes and changes in these outcomes are expressed as percent‐predicted unless otherwise stated. Participants were categorized according to their absolute change from baseline in FVC at Week 49 of the PAP using two definitions for improvement: first, > 0% predicted, to reflect any improvement; second, ≥ 3% predicted, to reflect the smallest improvement in FVC that a participant perceives as meaningful (see below). The 0% threshold is important from a clinical perspective, as any improvement in respiratory function may be considered a positive response to therapy given the presence of respiratory involvement prior to ERT start (FVC at baseline in the ALG arm of COMET: Median [range]: 62.5 [39–84]), and the expected decline in respiratory function characteristic of LOPD progression [14, 15]. In a sensitivity analysis, the 3% threshold was used as it corresponded to the within‐participant clinically meaningful threshold (CMT), derived by applying both anchor‐based (using patient‐reported outcomes as anchors) and distribution‐based analyses to the COMET data [16]. These analyses followed methodological guidelines [17, 18].

Urine samples obtained in a fasting state for the assessment of urinary glucose tetrasaccharide (HEX_4_) concentrations were collected at baseline (prior to ALG infusion) and longitudinally throughout the study to understand the effect of the switch to AVA on this glycogen burden biomarker.

Anti‐drug antibodies (ADAs) were also monitored to assess any association between change in FVC and development of ADAs (see Supporting Information, Appendix for details).

### 2.3 Statistical Modeling

Participants in the modified intention‐to‐treat (mITT) population who had received ALG in the PAP with at least one FVC value in each of the PAP and ETP were included in the analysis; data up to Week 145 of the ETP were included. Participants with missing change in FVC at week 49 were categorized as not meeting the definitions for improvement.

Piecewise linear mixed effects modeling was performed on the raw FVC expressed as percent‐predicted, using all timepoints from baseline to Week 145. This model included a continuous effect of time (weeks) and allowed separate pre‐ and post‐switch slopes for the time variable. This model also included ΔFVC status at Week 49 using the two definitions of improvement in FVC (> 0% and ≥ 3% predicted), with random intercept and slope at participant level. Separate models (%/year) were fit for the participant groups defined by ΔFVC status at Week 49 using the definitions listed above, with corresponding 95% confidence intervals (CI) and *p*‐values (*p*). Similar statistical modeling was used for the analysis of raw FEV1, MIP, and MEP, analyzed according to ΔFVC at Week 49.

Analyses on HEX4 concentrations and ADA were descriptive only.

No imputation of missing data was performed. All statistical analyses were conducted using SAS version 9.4.

## 3 Results

### 3.1 Study Participants and Baseline Characteristics

In the COMET trial, 49 participants with LOPD were randomized to receive ALG in the PAP [6]. Of these, 44 were included in the current study based on their agreement to enroll in the ETP, during which treatment was switched to AVA (Table S1). Of the 44 participants, 20 (45.5%), and 14 (31.8%) demonstrated ΔFVC > 0% and ΔFVC ≥ 3%, respectively, at the end of the PAP (*n* = 1 missing from the ALG cohort at Week 49). Key characteristics at baseline (start of PAP) were comparable between those achieving ΔFVC > 0% at Week 49 and those with ΔFVC ≤ 0% (Table 1); comparability was also seen between the groups defined by the 3% threshold (Table S2).

**TABLE 1:** Baseline characteristics of mITT population who received ALG during the PAP, stratified by ΔFVC at Week 49 (0% threshold).

Baseline characteristics	ALG Participants demonstrating ΔFVC > 0% (*n* = 20)	ALG Participants demonstrating ΔFVC ≤ 0% (*n* = 24)	Overall (*N* = 44)
Age (years)			
Median (range)	44.0 (26–70)	48.5 (19–77)	47.5 (19–77)
Sex, *n* (%)			
Male	11 (55)	13 (54)	24 (55)
Female	9 (45)	11 (46)	20 (45)
Race, *n* (%)			
White	20 (100)	23 (96)	43 (98)
Black or African American	0	1 (4)	1 (2)
Time from disease diagnosis (years)			
Median (range)	0.7 (0–27)	0.7 (0–18)	0.7 (0–27)
Time from first disease symptoms (years)			
Median (range)	10.6 (1–35)	8.5 (0–38)	10.0 (0–38)
FVC upright, % predicted			
Median (range)	58.3 (39–80)	63.2 (42–84)	62.5 (39–84)
FEV1, % predicted			
Median (range)	57.5 (42–83)	62.6 (42–82)	60.7 (42–83)
MIP, % predicted			
Median (range)	50.8 (18–107)	51.5 (23–234)	50.8 (18–234)
MEP, % predicted			
Median (range)	58.4 (20–126)	76.3 (28–201)	68.8 (20–201)
6MWT, % predicted			
Median (range)	54.7 (23–82)	55.8 (23–102)	55.8 (23–102)

*Note*: Participants with missing change from baseline in FVC were considered in the group of patients with a change from baseline ≤ 0%.

Abbreviations: 6MWT, 6‐min walking test; ALG, alglucosidase alfa; FVC, upright forced vital capacity; MEP, maximal expiratory pressure; MIP, maximal inspiratory pressure; mITT, modified intention‐to‐treat; PAP, primary analysis period; SD, standard deviation; ΔFVC, change in FVC at Week 49 of the PAP.

### 3.2 Trajectory of Respiratory Function During PAP and ETP Based on ΔFVC Status at Week 49

Spaghetti plots of FVC values were generated at the individual level at all timepoints and can be viewed in Figure S1 (0% threshold) and Figure S2 (3% threshold). One FVC outlier value at Week 97 for one participant was excluded, because the change from FVC at baseline (39.3%) to Week 97 (11.4%) was determined to be clinically implausible and recovered to a level similar to the pre‐Week 97 value at the next measurement.

Linear mixed effects modeling estimated slopes for evolution of respiratory function for groups characterized by ΔFVC status using the 0% and 3% thresholds are shown in Table 2. Among participants with ΔFVC > 0%, the estimated slope (SE) in FVC during the PAP was 4.0 (1.1) %/year (*p* < 0.01). Following switch to AVA in the ETP, FVC was maintained with an estimated slope (SE) not significantly different from zero: 0.1 (0.8) %/year (*p* = 0.86). For participants with ΔFVC ≤ 0%, the estimated slope (SE) in FVC while receiving ALG during the PAP was −3.4 (1.0) %/year (*p* < 0.01). Following switch to AVA in the ETP, FVC was stabilized with an estimated slope (SE) not significantly different from zero: 0.3 (0.7) %/year (*p* = 0.70).

**TABLE 2:** Estimated slopes of respiratory outcome measures in mITT population who received ALG during the PAP, stratified by ΔFVC at Week 49 (0% and 3% thresholds).

Slope of respiratory outcome	0% threshold for ΔFVC		3% threshold for ΔFVC	
	Participants demonstrating ΔFVC > 0% at Week 49 (*N* = 20)	Participants demonstrating ΔFVC ≤ 0% at Week 49 (*N* = 24)	Participants demonstrating ΔFVC ≥ 3% at Week 49 (*N* = 14)	Participants demonstrating ΔFVC < 3% at Week 49 (*N* = 30)
Slope of FVC in PAP (%/year)				
Estimate (SE)	4.0 (1.1)	−3.4 (1.0)	4.6 (1.3)	−2.1 (0.9)
95% CI	1.9, 6.1	−5.3, −1.4	2.1, 7.1	−3.8, −0.4
*p*	< 0.01	< 0.01	< 0.01	0.02
Slope of FVC in ETP (%/year)				
Estimate (SE)	0.1 (0.8)	0.3 (0.7)	0.3 (0.9)	0.2 (0.6)
95% CI	−1.4, 1.6	−1.1, 1.6	−1.6, 2.1	−1.1, 1.4
*p*	0.86	0.70	0.77	0.81
Slope of FEV1 in PAP (%/year)				
Estimate (SE)	4.3 (1.2)	−3.8 (1.1)	4.6 (1.5)	−2.2 (1.0)
95% CI	1.9, 6.8	−6.0, −1.5	1.6, 7.6	−4.2, −0.2
*p*	< 0.01	< 0.01	< 0.01	0.03
Slope of FEV1 in ETP (%/year)				
Estimate (SE)	−0.9 (0.9)	0.48 (0.8)	−1.0 (1.1)	0.2 (0.7)
95% CI	−2.6, 0.8	−1.1, 2.0	−3.2, 1.1	−1.2, 1.6
p	0.27	0.54	0.33	0.78
Slope of MIP in PAP (%/year)				
Estimate (SE)	3.5 (4.1)	−10.1 (3.8)	3.2 (5.0)	−7.1 (3.4)
95% CI	−4.5, 11.6	−17.6, −2.7	−6.5, 13.0	−13.8, −0.5
*p*	0.39	< 0.01	0.51	0.03
Slope of MIP in ETP (%/year)				
Estimate (SE)	3.5 (2.6)	3.2 (2.4)	4.2 (3.2)	2.9 (2.1)
95% CI	−1.6, 8.6	−1.5, 7.8	−2.1, 10.5	−1.2, 7.1
*p*	0.18	0.18	0.19	0.16
Slope of MEP in PAP (%/year)				
Estimate (SE)	6.8 (3.8)	0.8 (3.5)	8.2 (4.5)	1.3 (3.1)
95% CI	−0.6, 14.2	−6.0, 7.6	−0.7, 17.1	−4.7, 7.4
*p*	0.07	0.81	0.07	0.66
Slope of MEP in ETP (%/year)				
Estimate (SE)	6.4 (2.5)	3.1 (2.3)	8.2 (3.1)	3.0 (2.0)
95% CI	1.3, 11.4	−1.5, 7.7	2.1, 14.3	−1.1, 7.0
*p*	0.01	0.18	< 0.01	0.15

*Note*: Participants with missing change from baseline in FVC were considered in the group of patients with no improvement.

Abbreviations: CI, confidence interval; ETP; open‐label extended treatment period; FEV1, forced expiratory volume; FVC, upright forced vital capacity; MEP, maximal expiratory pressure; MIP, maximal inspiratory pressure; mITT, modified intention‐to‐treat; PAP, primary analysis period; SE, standard error. ΔFVC, change in FVC at Week 49 of the PAP.

**FIGURE 1:**
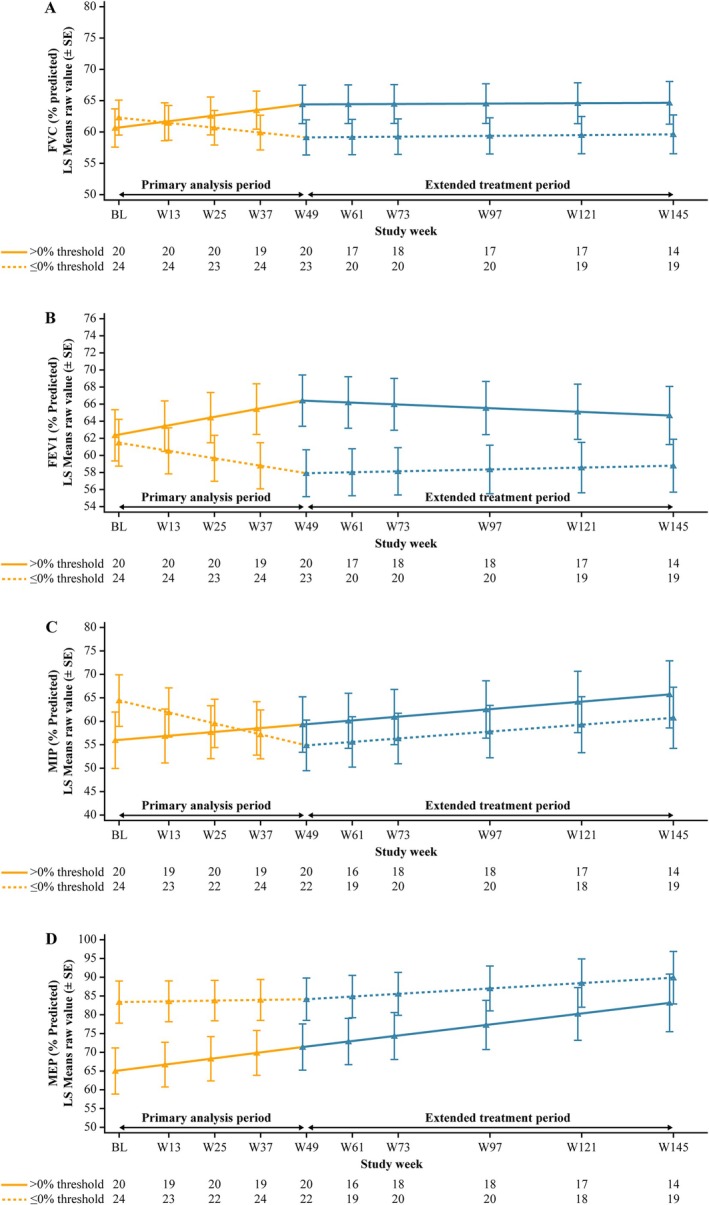
(A) FVC, (B) FEV1, (C) MIP, and (D) MEP. LS Means values over time (up to Week 145) in mITT population who received ALG during the PAP, stratified by ΔFVC (% predicted) from study baseline to Week 49 (0% threshold), based on piecewise linear mixed model. ALG, alglucosidase alfa; FEV1, percent‐predicted forced expiratory volume; FVC, percent‐predicted upright forced vital capacity; LS, least squares; MEP, percent‐predicted maximal expiratory pressure; MIP, percent‐predicted maximal inspiratory pressure; mITT, modified intention‐to‐treat; PAP, primary analysis period; SE, standard error; ΔFVC, absolute change in FVC at Week 49 of the PAP. Participants with missing change from baseline in FVC were considered in the group of patients with a change from baseline ≤ 0%.

The pattern of response for FEV1 matches the data shown for FVC, whereby changes in airflow during treatment with ALG were maintained after switch to AVA during the ETP. The patterns of response for MIP and MEP differed but, importantly, participants with ΔFVC < 0% showed stability in these measures of respiratory muscle strength following switch to AVA during the ETP (Table 2). Analysis of LS mean values of FVC, FEV1, MIP, and MEP over time (up to Week 145) are presented in Figure 1 and reinforce the modeled respiratory function slope data.

Overall, comparable results were observed in the sensitivity analysis using the 3% threshold (Table 2, Figure S3). Specifically, for participants with ΔFVC ≥ 3%, FVC and FEV1 increased with a trend towards increased MEP during treatment with ALG; these improvements were maintained or continued to increase after switching to AVA in the ETP. For participants with ΔFVC < 3%, declines in FVC, FEV1, and MIP were observed during ALG therapy, which stabilized after switching to AVA during the ETP.

### 3.3 Immunogenicity in Participants Defined by ΔFVC Status at Week 49

Analysis of the median anti‐ALG ADA peak titer in participants treated with ALG during the PAP was identical when comparing cohorts demonstrating ΔFVC > 0% versus ΔFVC ≤ 0% (1:3200 in both groups) (Table S3). Analysis of individual participant data showed that the proportion with anti‐ALG ADA peak titers ≥ 1:12 800 was higher in those with ΔFVC ≤ 0% (*n* = 10, 41.7%) than ΔFVC > 0% (*n* = 6, 30.0%). Similar patterns were observed when assessing participants demonstrating ΔFVC ≥ 3%, whereby the proportion with anti‐ALG ADA peak titers ≥ 1:12 800 was higher in participants with ΔFVC < 3% (*n* = 12, 40.0%) than ΔFVC ≥ 3% (*n* = 4, 28.6%) (data not shown). Analysis of ADA data during the ETP revealed that there were no participants with anti‐AVA ADA peak titers ≥ 1:12 800 (Table S3).

### 3.4 Pharmacodynamics in Participants Defined by ΔFVC Status at Week 49

Median (range) percent‐change from baseline in urinary HEX_4_ levels at Week 49 was −22.5% (−51.2%–102.8%) in participants with ΔFVC > 0% and −12.6% (−50.5%–93.6%) in those with ΔFVC ≤ 0% (Figure S4A). Following the switch to AVA in the ETP, HEX_4_ levels continued to decline up to Week 145 in both groups (Figure S4A). This trend was also observed when using the 3% threshold (Figure S4B).

## 4 Discussion

Stabilization of respiratory muscle function is an important treatment goal in LOPD due to the progressive and often irreversible lung function decline that can lead to the need for ventilation support and respiratory failure [19]. While some studies report stabilization of lung function for up to 5 years among those who initiate ALG treatment, other publications indicate that individuals may experience a gradual decline in vital capacity, particularly beyond 5 years [20]. This risk of further respiratory decline even with ALG treatment indicates a potential unmet need that might be addressed by alternative treatment options.

The COMET trial demonstrated clinical benefits of initiating AVA versus ALG in ERT‐naïve participants with LOPD [6]. This post hoc analysis of COMET data, including the ETP, shows that participants switching treatment from ALG to AVA experience beneficial outcomes regardless of prior treatment outcomes with ALG. Specifically, the improvement in respiratory function among participants with any improvement in FVC during the PAP was maintained after switching to AVA, while those who declined in FVC during the PAP experienced stabilization or improvement after switching to AVA. Similarly, levels of urine HEX_4_, a biomarker of glycogen burden, continued to decrease in the ETP. While it is possible that similar stabilization results may have been obtained if patients had remained on ALG, disease progression while receiving ALG has previously been reported in numerous studies and may, in part, be due to suboptimal uptake of ERT into skeletal muscle [21]. AVA was specifically designed to enhance uptake by skeletal muscle. This difference in design between ALG and AVA and the concordance of results across different respiratory outcomes (FVC, FEV1, MIP, and MEP) and across different definitions for meaningful change in lung function during ALG treatment (> 0% capturing clinical perspective and ≥ 3% capturing participant perspective) suggests that participants are likely to experience meaningful benefits after switching from ALG to AVA. Since the need for ventilatory support is closely linked to the level of respiratory dysfunction [20], the stabilization of FVC observed following the switch to AVA in this analysis would be expected to delay the need for either nocturnal or daytime respiratory support.

The present data showing stabilization of respiratory function following the switch from ALG to AVA represents a key improvement over prior data on long‐term treatment outcomes in Pompe disease, where 13‐year longitudinal real‐world data on ALG from the Pompe Registry showed a progressive decline in FVC (−0.54% to −1.0% predicted/yr) that started after 6 months of therapy [20]. Additional studies of up to 16‐year duration confirm a slow decline in FVC during ALG therapy (slope −1.0% to −4.6% predicted/yr) [3, 22, 23]. In contrast, long‐term data for AVA outcomes show stability of respiratory function for a 3‐year duration in COMET [13] and an 8‐year duration for the Neo1/Neo‐Ext studies [24]. Lastly, while there are limited data for patients who switch from ALG to AVA, two small studies also confirm stability of respiratory function for up to 6.5 years following the switch to AVA [21].

The ADA profile was similar across the two ΔFVC groups in the PAP, except for the incidence of titers > 1:12 800. Switching to AVA from ALG had minimal effect on the ADA profile.

### 4.1 Limitations

This is a post hoc analysis of a small group of patients with selected inclusion criteria. Of note, five participants randomized to receive ALG during the PAP did not enter the ETP. Furthermore, during the ETP, 14 enrolled participants discontinued the study, including five due to adverse events and eight who were lost to follow‐up, including one who officially withdrew from the study. Reasons for participant dropouts are unclear, and it is possible that missing data may have introduced bias into this analysis. However, the piecewise linear mixed effects modeling used to analyze the longitudinal trends in respiratory function minimizes this risk. Results from a broader group of patients and collected in real‐world settings may be helpful in confirming the generalizability of these findings. Additionally, the small sample size means conclusions cannot be drawn as to whether there is a correlation between ΔFVC and the development of ADA.

Prior definitions for meaningful change in ΔFVC (> 0% and ≥ 3%) were used in the present post hoc analysis. It is possible that participant perception of meaningful improvement varies according to baseline lung function. Given the sample size in COMET, further stratification by baseline FVC values was not deemed feasible, and this potential for differing thresholds was not considered in the current analysis.

## 5 Conclusions

Data from this post hoc analysis of people with LOPD enrolled in the COMET trial show clinically meaningful maintenance in measures of lung volume (FVC) and airflow (FEV1) after switching therapy from ALG to AVA that persists for at least 2 years and is independent of individual participants' level of benefit from prior treatment with ALG. The stabilization of respiratory function observed with long‐term treatment with AVA could potentially improve outcomes in people with LOPD by delaying the onset of respiratory failure and the need for mechanical ventilatory assistance. These results may be useful for people with LOPD and their healthcare providers to inform decision‐making on treatment options.

## Author Contributions

P.S.K., O.H.B., K.I.B., R.E.‐A., V.G., and N.S.R. made substantial contributions to study conception and design. P.S.K., J.D.M., and M.B. made substantial contributions to the acquisition of data. P.S.K., J.M., L.O., O.H.B., K.I.B., R.E.‐A., V.G., and N.S.R. made substantial contributions to analysis of data. P.S.K., J.M., L.O., J.D.M., S.W., K.I.B., V.G., and N.S.R. made substantial contributions to the interpretation of data. All authors were involved in drafting the work or revising it critically for important intellectual content and provided their final approval of the version to be published. All authors have agreed to be accountable for all aspects of the work in ensuring that questions related to the accuracy or integrity of any part of the work are appropriately investigated and resolved.

## Consent

This article reports post hoc analyses of the Phase 3 trial COMET trial (NCT02782741), for which informed consent was obtained.

## Conflicts of Interest

Jérôme Msihid: Employee of Sanofi and may hold stock and/or stock options in the company. Jordi Diaz‐Manera: Received funding for research from Sanofi, Boehringer, Sarepta, Astellas, Spark, and Amicus. Received honoraria as speaker or for consultancy from Sanofi, Sarepta, Lupin, Astellas, Spark, and Amicus. Kenneth I. Berger: Employee of Sanofi and may own stock or options in the company. Lasair O'Callaghan: Employee of Sanofi and may own stock or options in the company. Matthias Boentert: Received speaker honoraria and financial compensation for advisory board activities from Sanofi, Amicus, ITF, and Biogen. Has received a research grant from DGM (Deutsche Gesellschaft für Muskelkranke). Neeraj Singh Rawat: Employee of Sanofi and may own stock or options in the company. Olivier Huynh‐Ba: Employee of Sanofi and may own stock or options in the company. Priya S. Kishnani: Received research/grant support from Sanofi Genzyme and Amicus Therapeutics. She has received consulting fees and honoraria from Sanofi Genzyme, Amicus Therapeutics, and Asklepios Biopharmaceutical Inc. (AskBio). She is a member of the Pompe and Gaucher Disease Registry Advisory Board for Sanofi Genzyme, Pompe Disease Advisory Board for Amicus Therapeutics, and Advisory Board for Baebies. She has held equity in Asklepios Biopharmaceuticals and may receive milestone payments related to that equity in the future. Rachida Essadi‐Addou: Employee of Sanofi and may hold stock and/or stock options in the company. Stephan Wenninger: Received research grant by the DGM—Deutsche Gesellschaft für Muskelkranke e.V. and Sanofi‐Aventis Germany GmbH. He has served on advisory boards for Alexion Pharma, UCB Pharma GmbH, AMICUS Therapeutics, CSL Behring, Sanofi Genzyme GmbH, and ARGENX Therapeutics. He received funding for travel or speaker honoraria from Sanofi‐Aventis Germany GmbH; SH Glykogenose Gesellschaft; AbbVie Germany GmbH; Recordati Pharma GmbH; Diaplan GmbH; CSL Behring GmbH; Alexion Pharma GmbH; Desitin Germany; Akcea GmbH; AMICUS Therapeutics; and ARGENX Therapeutics. Victor Gallego: Employee of Sanofi and may own stock or options in the company.

## Supporting information


**Data S1.** Supporting Information.

## Data Availability

Qualified researchers may request access to participant‐level data and related study documents. Participantlevel data will be anonymized, and study documents will be redacted to protect the privacy of trial participants. Further details on Sanofi's data‐sharing criteria, eligible studies, and process for requesting access can be found at: https://www.vivli.org.
