# Supplementary material for: Efficacy of Switching Therapy From Alglucosidase Alfa to Avalglucosidase Alfa on Respiratory Function in Participants With Late‐Onset Pompe Disease: A Post Hoc Analysis From the COMET Trial
Source: JIMD Rep. 2025 Aug 12;66(5):e70033. doi: 10.1002/jmd2.70033 (PMC12343052; doi:10.1002/jmd2.70033)
Supplement: Supplementary file 1 — Data S1. Supporting Information. [file JMD2-66-e70033-s001.docx]

**Supplementary material for:**

**Efficacy of switching therapy from alglucosidase alfa to avalglucosidase alfa on respiratory function in participants with late-onset Pompe disease: a post-hoc analysis from the COMET trial**

# **Authors**

Priya S. Kishnani^1^, Matthias Boentert^2^, Stephan Wenninger^3^, Kenneth I. Berger^4^, Jérôme Msihid^5^, Lasair O’Callaghan^4^, Rachida Essadi-Addou^5^, Victor Gallego^6^, Neeraj Singh Rawat^7^, Olivier Huynh-Ba^5^, Jordi Diaz-Manera^8^

^1^Division of Medical Genetics, Department of Pediatrics, Duke University Medical Center, Durham, NC, USA.

^2^Department of Neurology and Institute of Translational Neurology, Münster University Hospital, Münster, Germany.

^3^LMU Clinic, Department of Neurology, Friedrich-Baur-Institute, University of Munich, Munich, Germany.

^4^Sanofi, Cambridge, USA.

^5^Sanofi, Gentilly, France.

^6^Sanofi, Madrid, Spain.

^7^Sanofi, Hyderabad, India.

^8^John Walton Muscular Dystrophy Research Centre, Newcastle University Centre for Life, Newcastle upon Tyne, UK; Neuromuscular Diseases Unit, Neurology Department, Hospital de la Santa Creu i Sant Pau, Barcelona, Spain; Centro de Investigación Biomédica en Red en Enfermedades Raras (CIBERER), Spain.

**Corresponding authors:** Kenneth I. Berger kenneth.berger@sanofi.com

*Table S1. FVC assessed in participants randomized to receive ALG in the PAP stratified by ΔFVC at Week 49*

| ΔFVC at Week 49 | Participants who completed PAP:  Alglucosidase alfa (N=49) | Participants who entered ETP:  Alglucosidase alfa (N=44) |
| --- | --- | --- |
| **Threshold of 0%, n (%)** |  |  |
| Participants above threshold | 20 (41) | 20 (45) |
| Participants below threshold | 29 (59) | 24 (55) |
| **Threshold of 3%, n (%)** | |  |
| Participants above threshold | 14 (29) | 14 (32) |
| Participants below threshold | 35 (71) | 30 (68) |

ALG, alglucosidase alfa; ETP, open-label extended treatment period; FVC, upright forced vital capacity; PAP, primary analysis period. ΔFVC, absolute change in FVC at Week 49 of the PAP. Participants with missing change from baseline in FVC were considered in the group of patients with a change from baseline ≤0%.

Table S2. Baseline characteristics of mITT population who received ALG during the PAP, stratified by ΔFVC at Week 49 (3% threshold)

| Baseline characteristics | Participants demonstrating  ΔFVC≥3% (n=14) | Participants demonstrating  ΔFVC<3%  (n=30) | Overall  (N=44) |
| --- | --- | --- | --- |
| **Age (years)** |  |  |  |
| Median (range) | 52.0 (26–70) | 46.5 (19–77) | 47.5 (19–77) |
| **Sex, n (%)** | | | |
| Male | 7 (50) | 17 (57) | 24 (55) |
| Female | 7 (50) | 13 (43) | 20 (45) |
| **Race, n (%)** | | | |
| White | 14 (100) | 29 (97) | 43 (98) |
| Black or African American | 0 | 1 (3) | 1 (2) |
| **Time from disease diagnosis (years)** | | | |
| Median (range) | 1.2 (0–27) | 0.7 (0–18) | 0.7 (0–27) |
| **Time from first disease symptoms (years)** | | | |
| Median (range) | 10.2 (1–29) | 9.9 (0–38) | 10.0 (0–38) |
| **FVC upright, % predicted** | | | |
| Median (range) | 58.3 (39–80) | 63.2 (42–84) | 62.5 (39–84) |
| **FEV1, % predicted** | | | |
| Median (range) | 57.5 (45–83) | 62.6 (42–82) | 60.7 (42–83) |
| **MIP, % predicted** | | | |
| Median (range) | 55.1 (18–105) | 48.3 (23–234) | 50.8 (18–234) |
| **MEP, % predicted** | | | |
| Median (range) | 58.05 (19.7–126.1) | 74.75 (27.5 –201.10 | 68.79 (19.7–201.1) |
| **6MWT, % predicted** | | | |
| Median (range) | 54.0 (23–82) | 55.8 (23–102) | 55.8 (23–102) |

6MWT, 6-minute walking test; ALG, alglucosidase alfa; FVC, percent-predicted upright forced vital capacity; MEP, maximal expiratory pressure; MIP, maximal inspiratory pressure; mITT, modified intention-to-treat; PAP, primary analysis period; SD, standard deviation; ΔFVC, change in FVC at Week 49 of the PAP. Participants with missing change from baseline in FVC were considered in the group of patients with a change from baseline ≤0%.

*Table S3: ADA for all participants in COMET who received ALG in PAP then switched to AVA in ETP, stratified by ΔFVC at Week 49 (0% threshold)*

| **ADA** | **Anti-ALG ADA response (PAP)** | | **Anti-AVA ADA response (ETP)** | |
| --- | --- | --- | --- | --- |
|  | **ΔFVC>0%  (N=20)** | **ΔFVC≤0% (N=28)** | **ΔFVC>0%  (N=20)** | **ΔFVC≤0% (N=24)** |
| ADA peak titer |  |  |  |  |
| Number, n | 20 | 24 | 17^a^ | 21^a^ |
| Median number of ADA peak titer (range) | 3200.0  (100–409,600) | 3200.0  (800–102,400) |  |  |
| Mean (SD) time to ADA peak titer from 1st infusion, weeks | 27.3 (16.0) | 27.5 (13.1) |  |  |
| ADA peak titer, n (%) |  |  |  |  |
| Negative | 0 | 0 | 0 | 0 |
| 100–800 | 3 (15) | 5 (21) | 3 (15) | 3 (13) |
| 1600–6400 | 11 (55) | 9 (38) | 0 | 0 |
| ≥12,800 | 6 (30) | 10 (42) | 0 | 0 |
| ADA peak titer ≥51,200, n (%) | 4 (20) | 4 (17) |  |  |
| Treatment emergent ADA^a^, n (%) |  |  | 14 (70) | 17 (71) |
| Treatment-induced ADA^b^, n (%) |  |  | 3 (100) | 3 (100) |
| Treatment-boosted ADA^c^, n (%) |  |  | 11 (65) | 14 (67) |

^a^ADA status positive at baseline; ^b^Treatment emergent ADA incidence is defined as 100 x (treatment boosted + treatment induced ADA positive patients)/(number of evaluable patients; ^c^Treatment induced ADA incidence is defined as 100 x (treatment induced ADA positive patients)/(number of evaluable patients with ADA negative at baseline); ^d^Treatment boosted ADA incidence is defined as 100 x (treatment boosted ADA positive patients)/(number of evaluable patients with ADA positive at baseline).

ADA, anti-drug antibodies; ALG, alglucosidase alfa; AVA, avalglucosidase alfa; ETP, open-label extended treatment period; FVC, upright forced vital capacity; PAP, primary analysis period; SD, standard deviation; ΔFVC, change in FVC at Week 49 of the PAP. Participants with missing change from baseline in FVC were considered in the group of patients with a change ≤0%.

*Figure S1. A: ΔFVC>0%, B: ΔFVC≤0%. Spaghetti plots of FVC over time (up to Week 289 in participants who received ALG in the PAP), stratified by ΔFVC at Week 49 using a threshold of 0%*


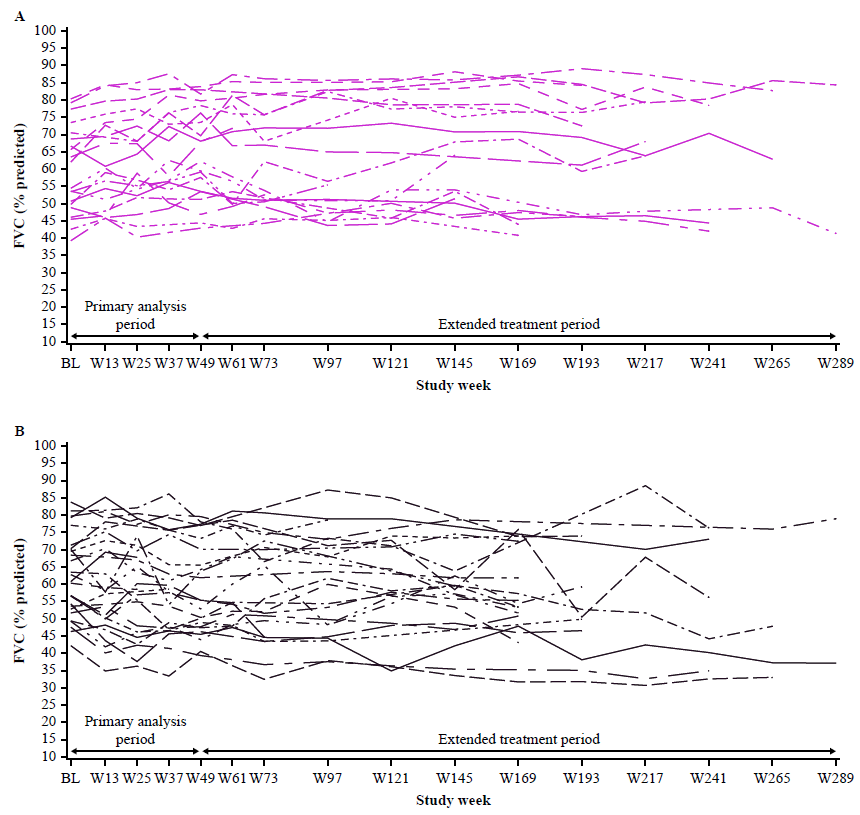


ALG, alglucosidase alfa; AVA, avalglucosidase alfa; ETP, open-label extended treatment period; FVC, upright forced vital capacity; PAP, primary analysis period; ΔFVC, change in FVC at Week 49 of the PAP. Participants with missing change from baseline in FVC were considered in the group of patients with a change ≤0%.

*Figure S2. A: ΔFVC≥3%, B: ΔFVC<3%. Spaghetti plots of FVC raw value over time (up to Week 289 in participants who received ALG in the PAP), stratified by ΔFVC at Week 49 using a threshold of 3%*


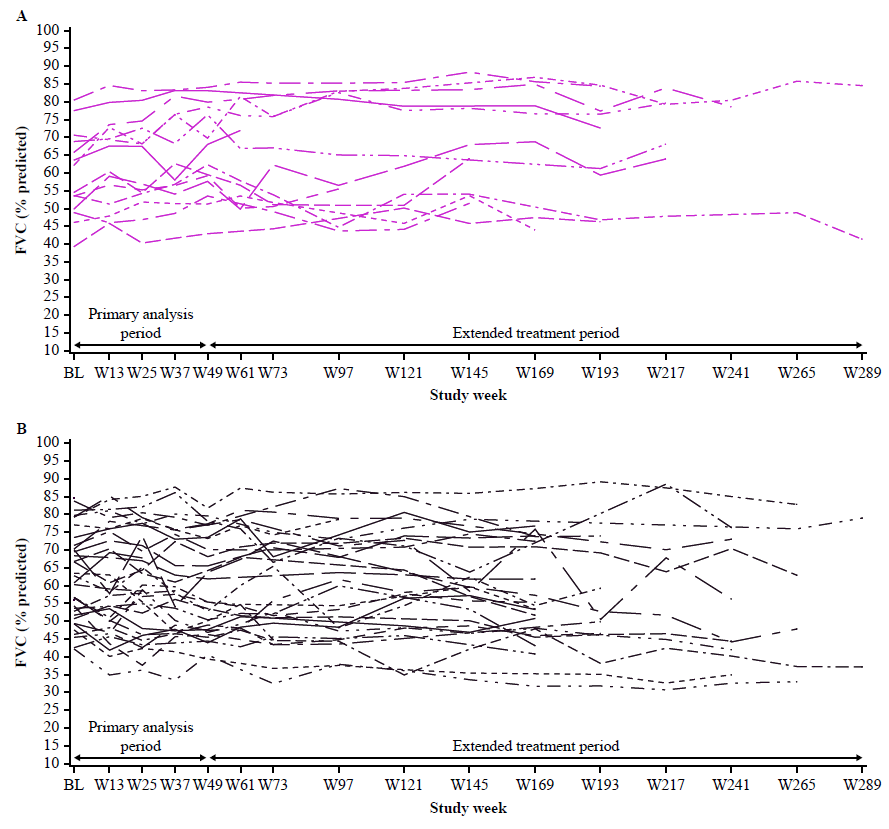


ALG, alglucosidase alfa; AVA, avalglucosidase alfa; ETP, open-label extended treatment period; FVC, upright forced vital capacity; PAP, primary analysis period; ΔFVC, change in FVC at Week 49 of the PAP. Participants with missing change from baseline in FVC were considered in the group of patients with a change ≤0%.

*Figure S3. A: FVC, B: FEV1, C: MIP and D: MEP. LS Means raw value over time (up to Week 145) in mITT population who received ALG during the PAP, stratified by ΔFVC (% predicted) from study baseline to Week 49 (3% threshold), based on piecewise linear mixed model.*


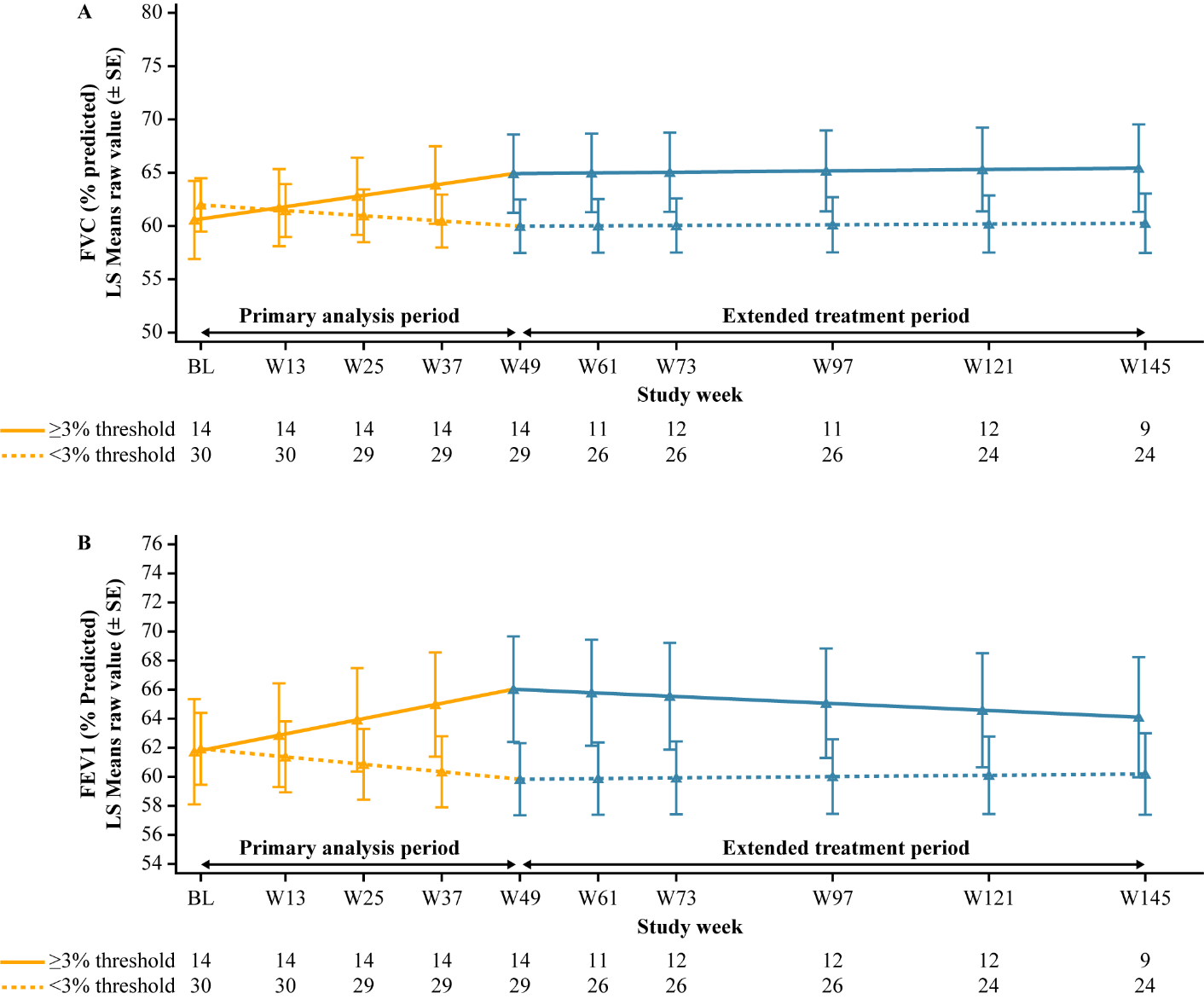


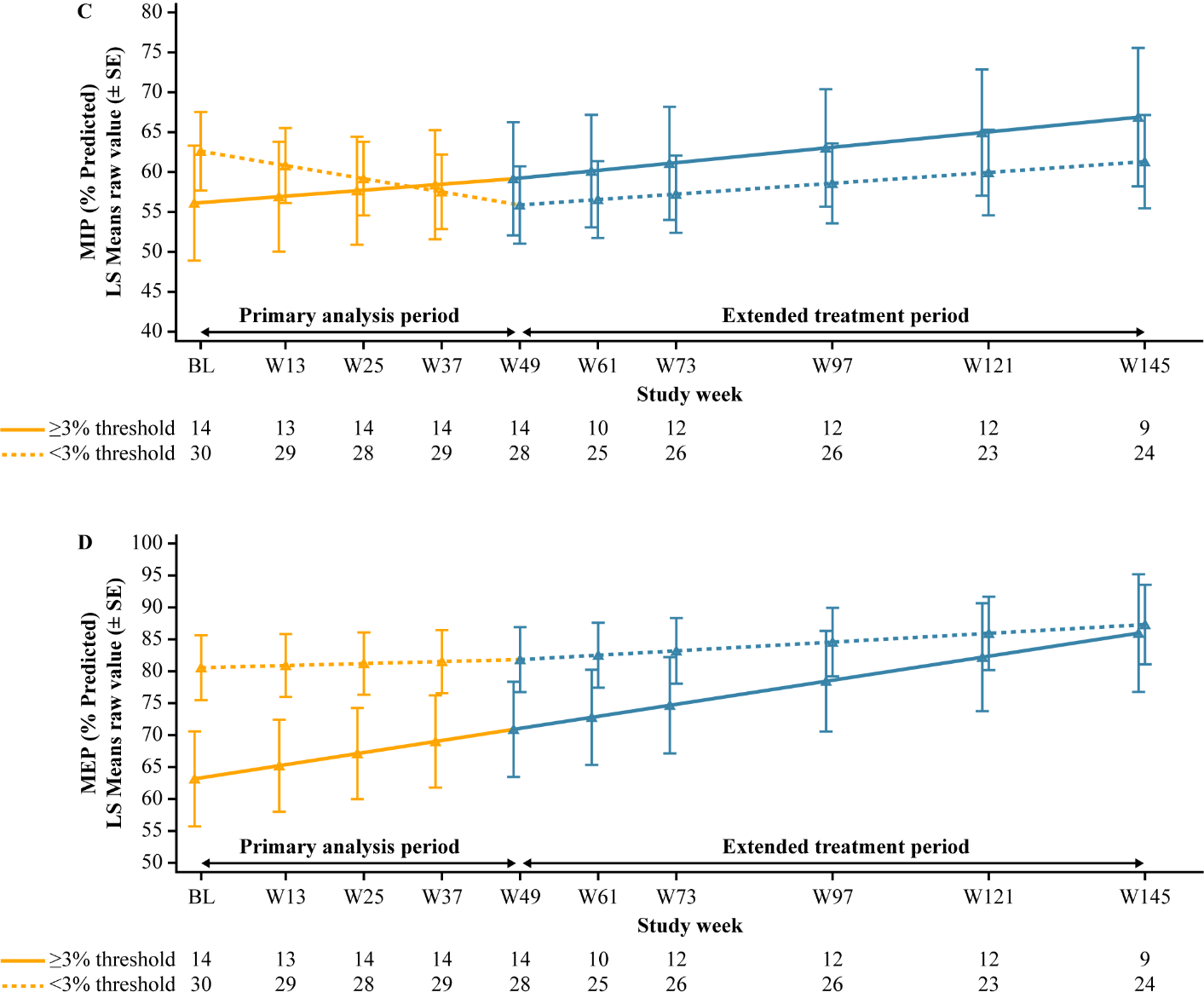


ALG, alglucosidase alfa; FEV1, forced expiratory volume; FVC, upright forced vital capacity; LS, least squares; mITT, modified intention-to-treat; MEP, maximal expiratory pressure; MIP, maximal inspiratory pressure; PAP, primary analysis period; SE, standard error. ΔFVC, change in FVC at Week 49 of the PAP. Participants with missing change from baseline in FVC were considered in the group of patients with a change from baseline ≤0%.

*Figure S4. A: 0% threshold, B: 3% threshold. Mean percent change (SE) from study baseline in urinary HEX4 levels over time stratified by ΔFVC at Week 49*


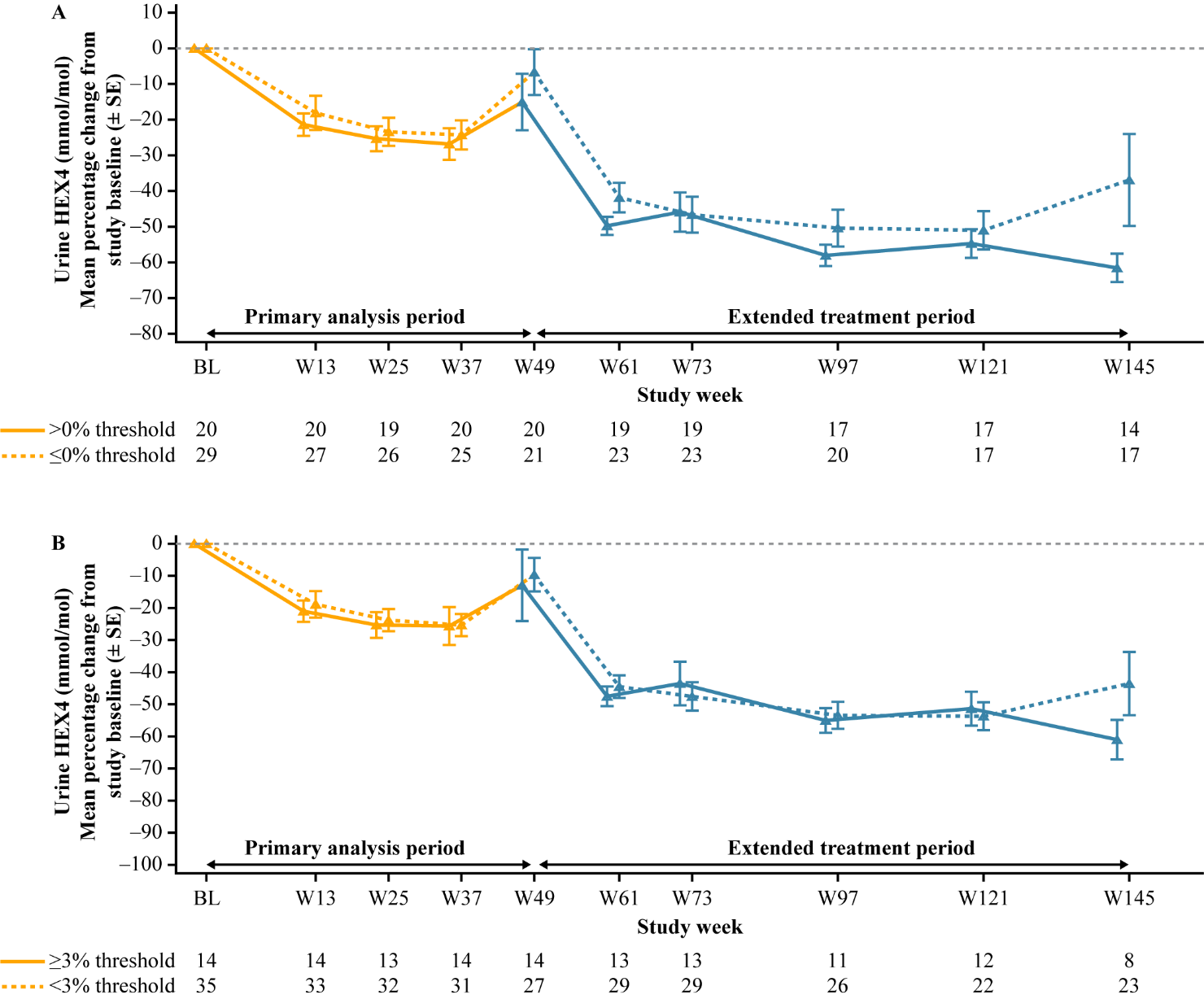


ALG, alglucosidase alfa; AVA, avalglucosidase alfa; FVC, upright forced vital capacity; HEX_4_, glucose tetrasaccharide; SE, standard error.

**Supplemental Appendix**

*Measurement of anti-drug antibodies (ADA)*

To understand the potential role that development of ADAs may have played in response to treatment, blood samples for evaluation of ADAs were collected from participants every month up to Week 73 and then every 3 months throughout the duration of the study. Samples were also collected from all participants at Weeks 2 and 52 to monitor for an early antibody response in participants treated with avalglucosidase alfa (AVA). ADA seropositive serum was assessed for neutralizing antibodies to AVA and/or alglucosidase alfa, as appropriate, including inhibition of enzyme activity and uptake. Samples were also evaluated for IgE, complement activation, and serum tryptase following moderate, severe, or recurrent mild infusion-associated reactions suggestive of hypersensitivity reactions.
